# Does prophylactic antibiotics post pediatric pyeloplasty reduce the incidence of febrile UTIs?

**DOI:** 10.1186/s12894-023-01301-x

**Published:** 2023-08-08

**Authors:** Kunj Sheth, Kathleen Puttmann, Paige Nichols, Jordon C. King, Huirong Zhu, Sheila Ryan, Andrew T. Gabrielson, Ming-Hsien Wang

**Affiliations:** 1 Baylor College of Medicine, Texas Children’s Hospital, Houston, TX USA; 2 Stanford University School of Medicine, Stanford Children’s Health, Stanford, CA USA; 3 Ohio State University College of Medicine, Columbus, OH USA; 4 Mayo Clinic Rochester, Rochester, MN USA; 5 Johns Hopkins University School of Medicine, 600 N Wolfe Street, Baltimore, MD 21287 USA

**Keywords:** Pediatrics, Pyeloplasty, Prophylactic Antibiotic (pAbx), Febrile Urinary Tract Infections (fUTI)

## Abstract

**Purpose:**

The use of postoperative prophylactic antibiotics in pediatric upper urinary tract reconstruction remains controversial. In this study, we examined whether low dose antibiotics administered following pediatric pyeloplasty reduce the incidence of febrile urinary tract infections at our institution. As a secondary outcome, in those patients with infection, additional analysis was performed to better quantify which patient population benefits the most from low dose prophylactic antibiotics.

**Methods:**

Institutional review board approval (IRB) was obtained. All methods were carried out in accordance with relevant guidelines and regulations. A retrospective study was performed in patients who underwent pyeloplasty (2011–2017) at our institution. Surgical approach (laparoscopic versus robotic assisted versus open, with or without internal JJ ureteral stent) were based on surgeon preference. Patients of 8 fellowship trained pediatric urologists were included in the study period. Patients with prior history of urologic interventions or other congenital genitourinary tract abnormalities were excluded. Demographics (age, gender, ethnicity, insurance status), prior history of culture proven urinary tract infection, surgical details (administration of perioperative antibiotics), and postoperative outcomes including; 1) re-admission 30 days post-surgery, 2) any urine cultures collected due to suspected urinary tract infection.

**Results:**

A total of 209 patients (149 boys, 60 girls) met our inclusion criteria with 55/209 (26%) receiving postoperative prophylactic antibiotics. The average age was 6 years (range: 2 months-18 years). Indwelling ureteral stent was used in 176 (84%) patients. Eleven patients (5%) had a culture-proven urinary tract infection within 30-days postoperatively. No significant differences were seen in postoperative complications or incidence of urinary tract infection when comparing surgical approaches, +/- ureteral stent, or the use of antibiotics. Secondary analysis noted statistically significant increase in post-operative urinary tract infection in younger children (2.8 v. 6.2 years, *p* = 0.02), those patients who had a positive preoperative urine culture (8/11, *p* = 0.01) and those with public health insurance (*p* = 0.038).

**Conclusion:**

The incidence of postoperative urinary tract infection following pyeloplasty in our cohort was relatively low. There was a higher incidence of urinary tract infection in patients less than 3 years old. The use of antibiotics in patients post pyeloplasty did not appear to affect the incidence of post-operative urinary tract infection, however, they may have a role in children who have not yet potty trained and in patients with positive preoperative urine culture.

## Background

The use of postoperative prophylactic antibiotics (pAbx) in pediatric upper urinary tract reconstruction remains controversial [1, 2]. With growing concern for the risk of early antibiotics exposure in the developing immune system [3, 4], we examined if the addition of pAbx post pediatric pyeloplasty reduces the incidence of febrile urinary tract infection (fUTIs) at our institution. In those children with a post-surgical fUTI, a secondary analysis was performed to evaluate which patient populations benefit the most from low dose pAbx.

## Methods

Ethical approval from the Institutional Review Board (IRB) was obtained. Inclusion criteria included patients 18 years or younger who underwent pyeloplasty between 2011 and 2017 at our institution, a major academic referral center. All methods were carried out in accordance with relevant guidelines and regulations. Per institutional policy, all patients received a single dose of IV antibiotics within 30 min prior to the start of the procedure, typically weight based Cephazolin unless they had a related allergy. In the event of an allergy, patients received Clindamycin. Patients receiving pAbx were prescribed Trimethoprim/Sulfamethoxazole unless they had a positive pre-operative culture that suggested use of another antibiotic. Patients were identified using the CPT codes 50400, 50405 (pyeloplasty), 50544 (laparoscopic), S2900 (robotic assisted). Decision regarding surgical approach, technique, stent placement, and pAbx were based on surgeon preference. The study included all pyeloplasties performed by eight ACGME fellowship trained pediatric urologists (> 3 years post training). Exclusion criteria included redo pyeloplasty, history of prior genitourinary interventions, solitary kidney, or any other congenital abnormalities of the genitourinary tract. Demographics (age, gender, ethnicity, and insurance status, surrogate for access to care), as well as prior antibiotic exposure unrelated to the urinary tract were collected. Any history of UTIs, type of perioperative Abx, intraoperative urine cultures, operative details, including surgical approach (open, laparoscopic or robot-assisted) and any intraoperative urinary drainage placement and post- surgery re-admissions were also collected. Postoperative fUTI was defined as a fever (> 38 °C) within 30 days of surgery, as well as positive urine culture (> 50,000 CFUs/ml) as defined by the 2011 AAP guideline [5]. Fisher exact test and Mann Whitney U test were used to compare patients with and without postoperative fUTIs. Statistical significance was defined as *p* value of < 0.05.

## Results

Of 255 patients who underwent pyeloplasty, 46 patients were excluded due to previously defined exclusion criteria. A total of 209 patients were included for analysis (Table 1). The average age was 6.0 years old (6.0 + / 5.1 years, 2 months old to 18 years old). In all, 149/209 (71%) patients in our study population were male and 83/209 (40%) had public insurance. Insurance status was used as a surrogate for access to care in our study. No patients in our study had a nephrostomy tube in place pre-operatively. In terms of surgical approach, 160/209 (77%) patients underwent robotic-assisted pyeloplasty, 31/209 (15%) had open repair, and 18/209 (9%) underwent conventional laparoscopic pyeloplasty without robotic-assistance. Intraoperative ureteral stents were placed in 176/209 (84%) of patients and 55/209 (26%) were discharged on pAbx. Thirty-five patients had an externalized drain placed, either in the form of a Penrose drain or a KISS (kidney internal splint/stent) drain. All internal stents were removed between 4–6 weeks postoperatively in the operating room with a single dose of preoperative prophylactic antibiotics. External drains were removed prior to discharge or within 7–10 days postoperatively.

**Table 1:** Patient demographics, treatment plan and outcome

	**Total (%)**	**Postoperative Febrile UTI (fUTI)**	***P* value**
Average Age	6.0 +/- 5.1 years	2.8 +/- 3.22 years	0.02
Gender:			
Male	149/209 (71%)	8/149	0.91
Female	60/209 (29%)	3/60	
Preopertive UCX:			
Positive	10/209 (5%)	3/10	0.01
Negative	199/209 (95%)	8/199	
Surgical Approach:			
Open	31/209 (15%)	0/31	0.23
Robotic	160/ 209 (77%)	11/160	
Laparoscopic	18/209 (9%)	0/18	
Drainage Method:			
Ureteral Stent	176/ 209 (84%)	11/ 176	0.22
External drain	35/ 209 (17%)	0/ 35	
Postoperative Antibiotics:			
Yes	55/209 (26%)	2/55	0.73
No	154/ 209 (74%)	9/154	
Insurance:			
Private	126/209 (60%)	5/126	0.038
Public (Medicaid)	83/209 (40%)	6/83	

In total, 11/209 (5%) patients experienced postoperative fUTIs per criteria. There was no statistical significance in terms of surgical approach, presence of stent, or the use of pAbx in the 11 patients who presented with postoperative fUTI. There was a statistical significance noted in; 1) the age of the patients (2.8 +/- 3.22 years, *p* = 0.02), and 2) those with positive preoperative urine culture (10/209, *p* = 0.01). Of note those with positive preoperative urine cultures were asymptomatic at the time of culture collection and these patients were treated with culture-directed antibiotics. On a sub-group analysis, we found that patients with public insurance (Medicaid) were more likely to have a culture-proven fUTI compared to those without public insurance (Table 1, *p* = 0.038).

## Discussion

Among children undergoing routine pyeloplasty, the incidence of postoperative fUTI in our cohort was relatively low (5%), consistent with current reported rates of 1–3% [6, 7]. When evaluating potential risk factors for development of fUTI in the initial 30-day postoperative period, we did not identify an association between the use of pAbx, surgical approach, or urinary stent placement. However, younger age and the presence of a positive culture within the month to surgery did correlate with a higher incidence of fUTI. Furthermore, sub-group analysis indicates that insurance status might also impact the presentation of fUTIs. Similar results were seen in Ferroni et al., where postoperative antibiotics did not alter the rate of UTIs in patients undergoing minimally-invasive pyeloplasty [8]. We had total of 11 out of 209 patients with fUTI post-operatively, all eleven had robotic assisted approach (11/209). One possibility is that robotic approach is the most common surgical approach in our institution (160/209, 77%).

While our study indicates that pAbx does not impact the incidence of post-pyeloplasty fUTIs in majority of our patients, there is a selective group of young children who might benefit from pAbx. Our analysis indicates that children younger than 3 years old have a higher risk of post- operative fUTIs. This might be related to toilet-training, similar to the population included in the 2011 AAP UTI guideline [5].

While there has been concern for potential infection risk associated with ureteral stent usage due to high colonization rates [9, 10], the risk of infection is low when they are maintained for less than 90 days [10]. Since our post-pyeloplasty stents remain in place for at most 4–6 weeks, it makes sense that we did not find any association between use of a ureteral stent and postoperative fUTI. We also used patient insurance type, public (Medicaid) versus private, as a surrogate for potential socioeconomic status. In our cohort, patients with public insurance, we noted an increase incidence of fUTI. Future study will help elucidate factors, such as language, location, and availability to health care providers as potential barriers.

As we hypothesized earlier, children pre-potty training might be at a higher risk of fUTI post upper urinary tract reconstruction. One additional factor common to young patients is the increase incidence of upper respiratory tract infections (URIs). Studies have shown that fUTI is more commonly associated with children diagnosed with bronchiolitis [11–14]. The current AAP recommendation on children with URIs, is to treat if there are symptoms concerning for UTIs as well [14]. Based on these studies, it may be prudent to obtain a preoperative urine culture within 30 days of surgery for patients under 2 years of age with a history of multiple URIs.

Our study was limited by its retrospective nature. The patients were all evaluated, treated, and followed at a major referral academic children’s center. Our results may not be applicable to the general community. In addition, robotic-assisted pyeloplasty encompassed 77% of our study population. Robotic surgical systems and pediatric-specific instrumentation may not always be available. Another limitation is we were unable to assess what percentage of the male patients were circumcised which could contribute to fUTI rate. Although the decision to prescribe pAbx was surgeon-specific and was considered irrespective of case features, select patients may have been started on pAbx because of high-risk features which could contribute to selection bias. In order to be certain on the utility of pAbx post urinary tract reconstruction, multi-institutional collaboration with systematic follow up are needed to better define the true benefits of prophylactic antibiotics.

## Conclusion

Overall, our data show no increased risk of postoperative fUTIs with use of ureteral stents, or addition of pAbx in children undergoing pyeloplasty. However, judicious use in selected high- risk patients, such as those with positive cultures within 30 days of surgery and those that have not yet been potty trained, might lower the risk of fUTI. Further studies prospective, multi-institutional studies will better delineate a pAbx treatment algorithm for patients undergoing pyeloplasty.

## Data Availability

The datasets used during the current study are available from the corresponding author on reasonable request.

## Abbreviations

pAbx: Prophylactic antibiotics
fUTI: Febrile urinary tract infection
IRB: Institutional Review Board
